# Synthesis and structure of (*Z*)-2-(eth­oxy­methyl­idene)-2,3,4,9-tetra­hydro-1*H*-carbazol-1-one

**DOI:** 10.1107/S2056989026008169

**Published:** 2026-08-14

**Authors:** Makuteswaran Sridharan, Aravazhi Amalan Thiruvalluvar

**Affiliations:** a Department of Chemistry, RV College of Engineering, Bangalore 560 059, Karnataka, India; b Principal (Retired), 63 Shanthi Nagar, 5th Street, Nanjikottai Road, Thanjavur 613 006, Tamilnadu, India; University of Aberdeen, United Kingdom

**Keywords:** crystal structure, carbazol-1-one, C—H⋯O and N—H⋯O hydrogen bonds, C—H⋯π and π–π contacts

## Abstract

The title compound, C_15_H_15_NO_2_, crystallizes with two independent mol­ecules (*A* and *B*) in the asymmetric unit. One of the methyl­ene C atoms in the cyclo­hexene ring in mol­ecule *A* was refined as disordered over two sites in a 0.818 (5) to 0.182 (5) ratio. In the crystal, pairwise N—H⋯O hydrogen bonds generate centrosymmetric *A* + *A* and *B* + *B* dimers characterized by *R*^2^_2_(10) loops and the dimers are linked by *A* + *B* and *B* + *A* C—H⋯O and C—H⋯π inter­actions. Aromatic π–π stacking inter­actions are also present and together, the inter­molecular inter­actions generate a three-dimensional network.

## Abstract 2

Two crystallographically independent mol­ecules are present in the asymmetric unit of the title compound. C—H⋯O, N—H⋯O, C—H⋯π and π–π contacts connect mol­ecules in the crystal.

## 1. Chemical context

Carbazole derivatives continue to occupy a central position in heteroaromatic chemistry with recent investigations underscoring their synthetic versatility and broad pharmacological potential. In particular, 2,3,4,9-tetra­hydro­carbazol-1-ones have emerged as valuable precursors for the construction of diverse heterocyclic frameworks, a theme reinforced by our recent contributions (Sridharan *et al.*, 2026 ▸; Sridharan & Thiruvalluvar, 2026*a* ▸,*b* ▸). The eth­oxy­methyl­idene substituent at the 2-position introduces an electrophilic center that readily undergoes condensation and cyclization, while the carbonyl group at the 1-position enhances reactivity and synthetic adaptability. This dual functionality reflects current trends in heterocyclic design, where multi-reactive scaffolds are increasingly favored for pharmaceutical applications. Parallel to these synthetic advances, carbazole derivatives have been extensively investigated for their anti­cancer, anti-inflammatory and anti­parasitic activities, confirming their broad therapeutic relevance. As part of our studies in this area, we now describe the synthesis and structure of the title compound, C_15_H_15_NO_2_ (**I**).
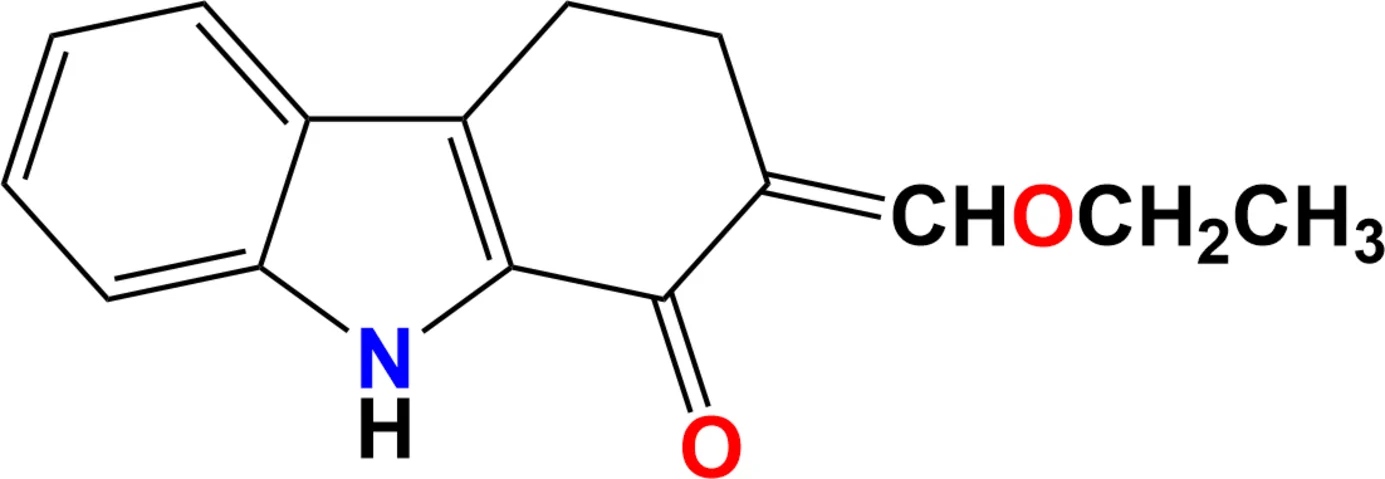


## 2. Structural commentary

**Figure 1:**
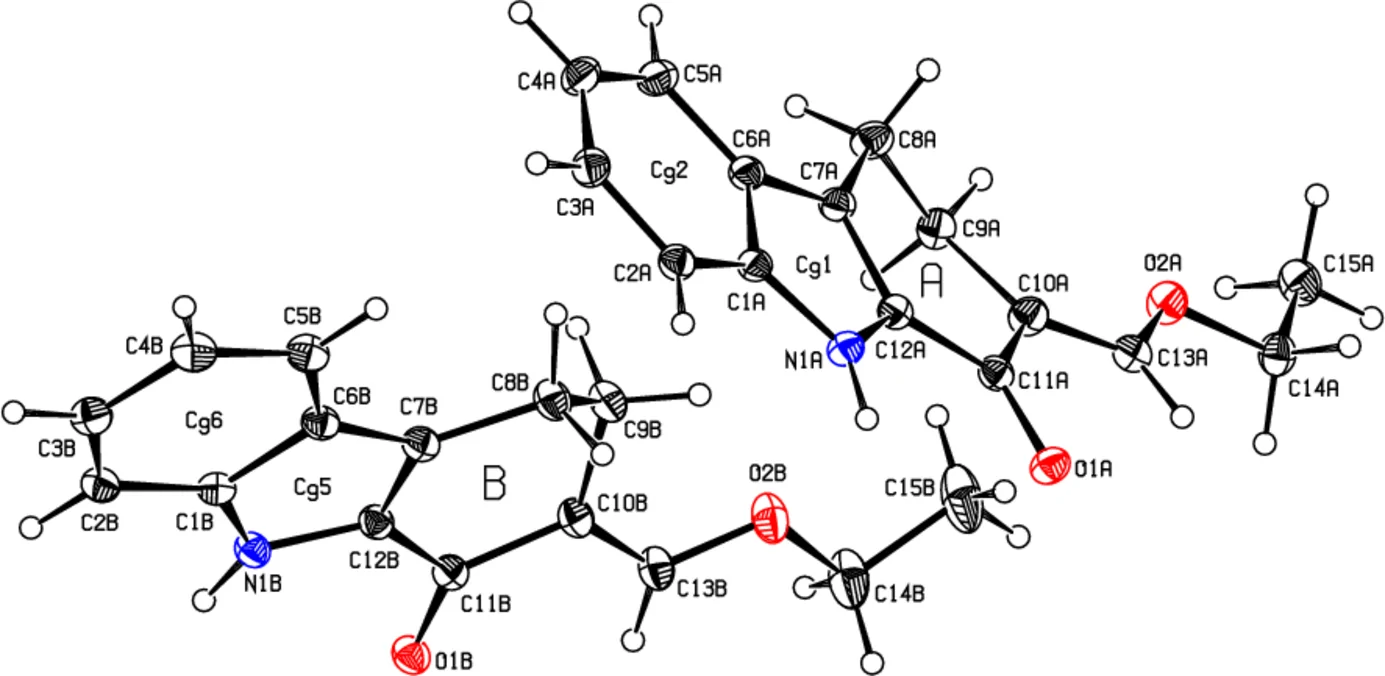
The mol­ecular structure of (**I**), showing displacement ellipsoids drawn at the 30% probability level.

As shown in Fig. 1 ▸, compound (**I**), which crystallizes in the triclinic space group *P*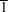 with two mol­ecules (*A* and *B*) in the asymmetric unit, consists of indole and cyclo­hexene units fused *via* the C7*A*—C12*A* and C7*B*—C12*B* bonds. The methylene carbon atom C9 in molecule *A* is disordered over two sites (C9*A* and C9*C*) with refined occupancies of 0.818 (5) and 0.182 (5), respectively, and only the major component C9*A* is considered in the following discussion. As expected, the pyrrole (N1/C1/C6/C7/C12) and benzene (C1–C6) rings are nearly co-planar, subtending a dihedral angle of 1.25 (7)° in mol­ecule *A* and 1.11 (7)° in mol­ecule *B*. A puckering analysis (Cremer & Pople, 1975 ▸) of ring *A* (C7A—C12A) gave the parameters *q*_2_ = 0.2996 (16) Å, *q*_3_ = −0.1970 (16) Å, *Q*_T_ = 0.3586 (18) Å, θ = 123.3 (3)° and φ = 292.6 (3)°, which corresponds to an envelope conformation, where atom C9*A* (part of the methyl­ene group adjacent to the exocyclic C=C double bond) is at the flap position and displaced by 0.5020 (24) Å away from the best plane of the remaining atoms. A similar analysis for ring *B* (C7*B*–C12*B*) gave *q*_2_ = 0.3342 (15) Å, *q*_3_ = −0.1831 (15) Å, *Q*_T_ = 0.3810 (16) Å, θ = 118.7 (2)° and φ = 284.4 (3)°, indicating an envelope conformation, where atom C9*B* is at the flap position and −0.5175 (19) Å away from best plane of the remaining atoms. The C7*A*—C8*A*—C9*A*—C10*A* torsion angle in ring *A* is −40.90 (18)° and the C7*B*—C8*B*—C9*B*—C10*B* torsion angle in ring *B* is −43.16 (16)°. The eth­oxy­methyl­ene side chain in mol­ecule A adopts an extended conformation, as indicated by the C10*A*—C13*A*—O2*A*—C14*A* and C13*A*—O2*A*—C14*A*—C15*A* torsion angles of −177.10 (12) and −178.88 (11)°, respectively. Similarly, the eth­oxy­methyl­ene side chain in mol­ecule *B* also adopts an extended conformation, as indicated by the C10*B*—C13*B*—O2*B*—C14*B* and C13*B*—O2*B*—C14*B*—C15*B* torsion angles of −179.32 (14) and 173.13 (15)°, respectively.

## 3. Supra­molecular features

**Figure 2:**
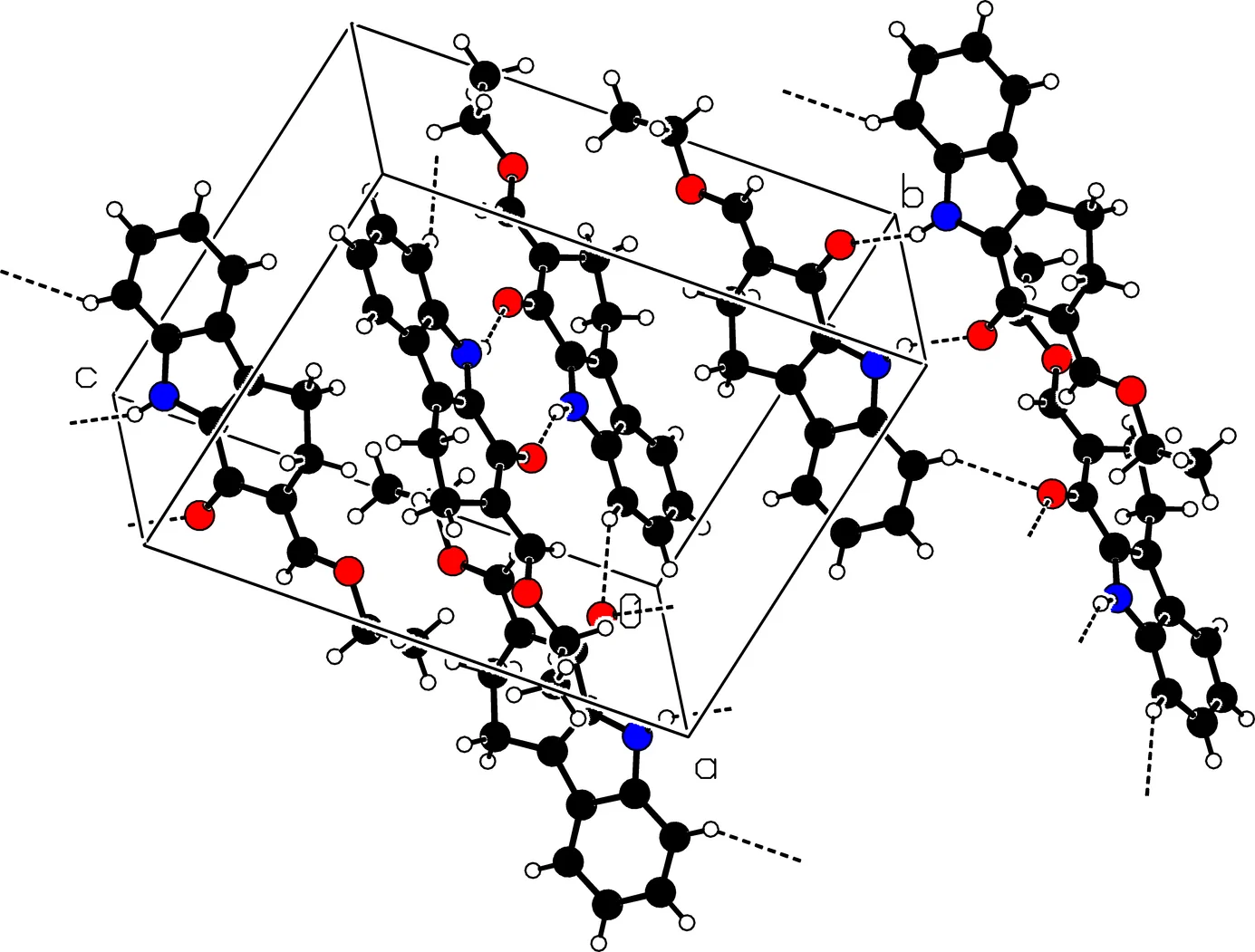
Partial packing view of (**I**), in minimum view direction, showing the hydrogen bonds. Black dashed lines represent C—H⋯O and N—H⋯O hydrogen bonds.

**Figure 3:**
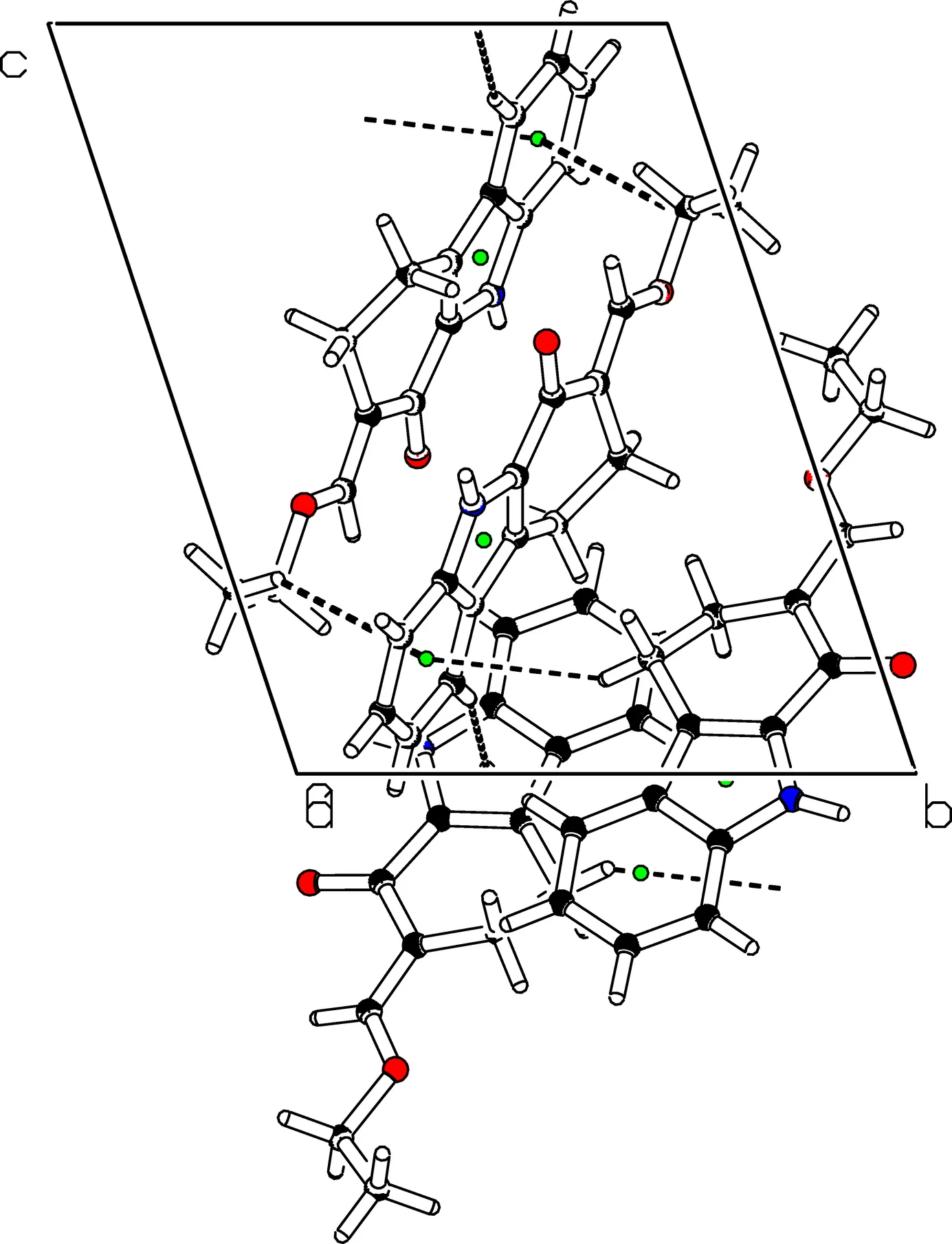
Straw-style packing view of (**I**), viewed down the *a-*axis direction, showing the C—H⋯π contacts. Centroids are shown as green spheres and black dashed lines are H⋯π contacts.

**Figure 4:**
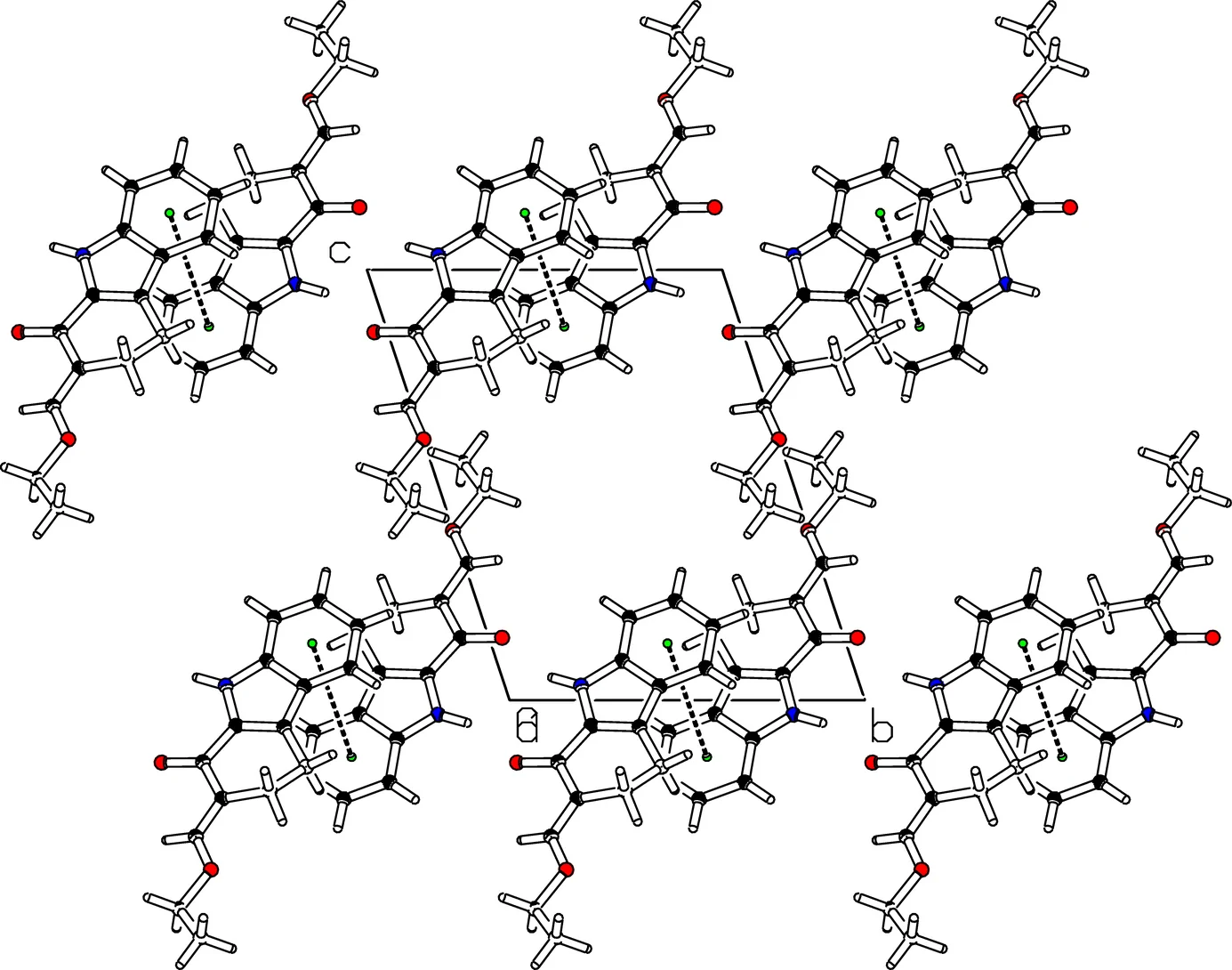
The straw-style crystal structure of (**I**), showing the formation of π–π stacking inter­actions [Symmetry code: 1 − *x*, 1 − *y*, −*z*]. Centroids are given as green spheres and black dashed lines are the π–π contacts.

In the extended structure of (**I**), the mol­ecules are connected by C—H⋯O (*A*⋯*B* and *B*⋯*A* pairs) and N—H⋯O (*A*⋯*A* and *B*⋯*B* pairs) hydrogen bonds (Table 1 ▸; Fig. 2 ▸). Both crystallographically independent mol­ecules are linked by similar N1*A*—H1*A*⋯O1*A*(1 − *x*, 1 − *y*, 1 − *z*) and N1*B*—H1*B*⋯O1*B*(1 − *x*, 2 − *y*, −*z*) hydrogen bonds thereby forming centrosymmetric 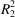
(10) loops. Weak C2*A*—H2*A*⋯O1*B*(*x*, −1 + *y*, *z*) and C2*B*—H2*B*⋯O1*A*(*x*, *y*, −1 + *z*) hydrogen bonds are also present. The mol­ecules are further linked by three C—H⋯π inter­actions, *viz*.: C5*A*—H5*A*⋯*Cg*5(−*x*, 1 − *y*, −*z*), C8*B*—H8*D*⋯*Cg*2(*x*, *y*, *z*), and C15*A*—H15*A*⋯*Cg*2(−*x*, 1 − *y*, 1 − *z*), where *Cg*5 and *Cg*2 are the centroids of the (N1*B*/C1*B*/C6*B*/C7*B*/C12*B*) pyrrole ring and (C1*A*–C6*A*) benzene ring (Fig. 3 ▸). A π–π stacking contact is also observed (Fig. 4 ▸) with the distance between the ring centroids *Cg*6⋯*Cg*6 (1 − *x*, 1 − *y*, −*z*) being 3.9540 (8) Å where *Cg*6 is the centroid of the (C1*B*–C6*B*) ring.

## 4. Database survey

A search of the Cambridge Structural Database (CSD, Version 6.01, updated to February 2026; Groom *et al.*, 2016 ▸) using the core structure of (**I**) gave zero hits. Database searches were performed using CONQUEST, and structural analyses were carried out using *Mercury* (Macrae *et al.*, 2020 ▸).

## 5. Synthesis and crystallization

**Figure 5:**
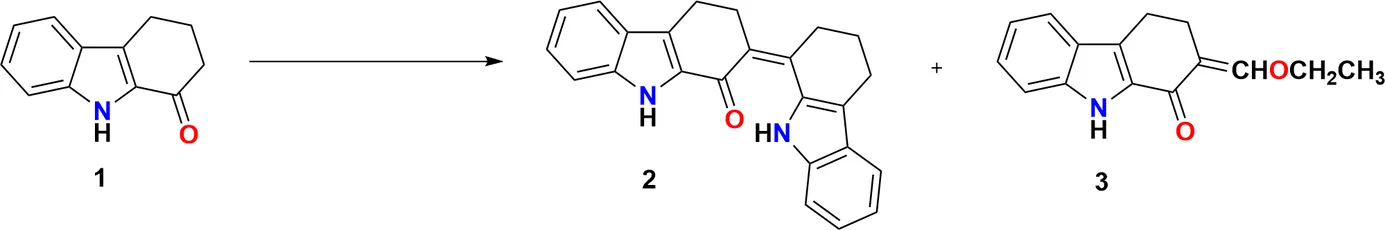
The synthesis scheme for (**I**).

2,3,4,9-Tetra­hydro­carbazol-1-one (**1**, 0.005 mol) in di­chloro­methane (15 ml) was added to an ice-cooled solution of di­eth­oxy­carbenium fluoro­borate [prepared *in situ* from BF_3_·Et_2_O (1.65 ml; 0.01 mol) and HC(OEt_3_) (1.25 ml; 0.01 mol)]. The reaction mixture was kept at 258–263 K. To this mixture, tri­ethyl­amine (0.01 mol) was added dropwise and the stirring was continued over a period of five h. The reaction was monitored by TLC. After the completion of reaction, the excess solvent was then removed and extracted using ethyl acetate dried over anhydrous sodium sulfate. The resulting brown solid was then separated by column chromatography over silica gel using petroleum ether: ethyl acetate as eluants (99:1) and (95:5) to yield 2,3,4,9-tetra­hydro-2-(2′,3′,4′,9′-tetra­hydro­carbazol-1-yl­idene)-carbazol-1-one (**2**) and (*Z*)-2-(eth­oxy­methyl­idene)-2,3,4,9-tetra­hydro­carbazol-1-one (**3**). The chemical structures of the final products were confirmed by NMR spectroscopy and elementary analysis data. Compound (**3**) was recrystallized from ethanol solution as yellow prisms (0.789 g, 70%), m.p. 410–412 K. The reaction scheme is shown in Fig. 5 ▸.

## 6. Refinement

Crystal data, data collection and structure refinement details are summarized in Table 2 ▸. The N-bound H atoms (H1*A* and H1*B*) were located in a difference-Fourier map and their positions were freely refined with *U*_iso_(H) = 1.2*U*_eq_(N). All the other H atoms were placed in calculated positions and refined as riding atoms with *U*_iso_(H) = 1.2*U*_eq_(C) or 1.5*U*_eq_(methyl C). The methyl group was allowed to rotate, but not to tip, to best fit the experimental electron density. One of the methyl­ene C atoms in the cyclo­hexene ring in mol­ecule *A* was refined as disordered over two sites. The occupancy ratio refined to C9*A*:C9*C* = 0.818 (5):0.182 (5).

## Supplementary Material

Crystal structure: contains datablock(s) I. DOI: 10.1107/S2056989026008169/hb8246sup1.cif

Structure factors: contains datablock(s) I. DOI: 10.1107/S2056989026008169/hb8246Isup2.hkl

Supporting information file. DOI: 10.1107/S2056989026008169/hb8246Isup3.cdx

Supporting information file. DOI: 10.1107/S2056989026008169/hb8246Isup4.cml

CCDC reference: 1540677

Additional supporting information:  crystallographic information; 3D view; checkCIF report

## Figures and Tables

**Table 1:** Hydrogen-bond geometry (Å, °) *Cg*5 and *Cg*2 are the centroids of the (N1*B*/C1*B*/C6*B*/C7*B*/C12*B*) pyrrole ring and (C1*A*–C6*A*) benzene rings respectively.

*D*—H⋯*A*	*D*—H	H⋯*A*	*D*⋯*A*	*D*—H⋯*A*
C2*A*—H2*A*⋯O1*B*^i^	0.95	2.54	3.3335 (16)	141
C2*B*—H2*B*⋯O1*A*^ii^	0.95	2.54	3.3457 (16)	142
N1*A*—H1*A*⋯O1*A*^iii^	0.919 (16)	1.985 (16)	2.8462 (14)	155.4 (13)
N1*B*—H1*B*⋯O1*B*^iv^	0.908 (17)	2.017 (17)	2.8807 (14)	158.5 (14)
C5*A*—H5*A*⋯*Cg*5^v^	0.95	2.66	3.5939 (16)	166
C8*B*—H8*D*⋯*Cg*2	0.99	2.97	3.7871 (16)	141
C15*A*—H15*A*⋯*Cg*2^vi^	0.98	2.89	3.6449 (18)	134

Symmetry codes: (i) 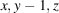; (ii) 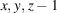; (iii) 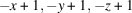; (iv) 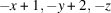; (v) 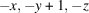; (vi) 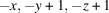.

**Table 2:** Experimental details

Crystal data	
Chemical formula	C_15_H_15_NO_2_
*M*_r_	241.28
Crystal system, space group	Triclinic, *P* 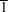
Temperature (K)	100
*a*, *b*, *c* (Å)	10.1536 (5), 10.2760 (5), 13.5207 (6)
α, β, γ (°)	103.632 (1), 107.867 (1), 100.974 (1)
*V* (Å^3^)	1251.14 (10)
*Z*	4
Radiation type	Mo *K*α
μ (mm^−1^)	0.09
Crystal size (mm)	0.54 × 0.48 × 0.41

Data collection	
Diffractometer	Bruker SMART APEX CCD diffractometer
Absorption correction	Multi-scan (*SADABS2004*; Krause *et al.*, 2015 ▸)
*T*_min_, *T*_max_	0.912, 0.966
No. of measured, independent and observed [*I* > 2σ(*I*)] reflections	13043, 6179, 5170
*R*_int_	0.021
(sin θ/λ)_max_ (Å^−1^)	0.667

Refinement	
*R*[*F*^2^ > 2σ(*F*^2^)], *wR*(*F*^2^), *S*	0.048, 0.129, 1.07
No. of reflections	6179
No. of parameters	342
H-atom treatment	H atoms treated by a mixture of independent and constrained refinement
Δρ_max_, Δρ_min_ (e Å^−3^)	0.34, −0.21

Computer programs: *SMART* (Bruker, 2002 ▸) and *SAINT-Plus* (Bruker, 2003 ▸), *SHELXS* (Sheldrick, 2008 ▸), *SHELXL2025/1* (Sheldrick, 2015 ▸), *PLATON* (Spek, 2020 ▸) and *publCIF* (Westrip, 2010 ▸).

## supplementary crystallographic information

### (Z)-2-(Ethoxymethylidene)-2,3,4,9-tetrahydro-1H-carbazol-1-one Crystal data

**Table:**

C_15_H_15_NO_2_	*Z* = 4
*M**_r_* = 241.28	*F*(000) = 512
Triclinic, *P*1	*D*_x_ = 1.281 Mg m^−^^3^
*a* = 10.1536 (5) Å	Mo *K*α radiation, λ = 0.71073 Å
*b* = 10.2760 (5) Å	Cell parameters from 8496 reflections
*c* = 13.5207 (6) Å	θ = 2.2–30.6°
α = 103.632 (1)°	µ = 0.09 mm^−^^1^
β = 107.867 (1)°	*T* = 100 K
γ = 100.974 (1)°	Block, yellow
*V* = 1251.14 (10) Å^3^	0.54 × 0.48 × 0.41 mm

### (Z)-2-(Ethoxymethylidene)-2,3,4,9-tetrahydro-1H-carbazol-1-one Data collection

**Table:**

Bruker SMART APEX CCD diffractometer	6179 independent reflections
Radiation source: fine-focus sealed tube	5170 reflections with *I* > 2σ(*I*)
Graphite monochromator	*R*_int_ = 0.021
ω scans	θ_max_ = 28.3°, θ_min_ = 2.2°
Absorption correction: multi-scan (SADABS2004; Krause *et al.*, 2015)	*h* = −13→13
*T*_min_ = 0.912, *T*_max_ = 0.966	*k* = −13→13
13043 measured reflections	*l* = −18→18

### (Z)-2-(Ethoxymethylidene)-2,3,4,9-tetrahydro-1H-carbazol-1-one Refinement

**Table:**

Refinement on *F*^2^	Primary atom site location: structure-invariant direct methods
Least-squares matrix: full	Secondary atom site location: difference Fourier map
*R*[*F*^2^ > 2σ(*F*^2^)] = 0.048	Hydrogen site location: mixed
*wR*(*F*^2^) = 0.129	H atoms treated by a mixture of independent and constrained refinement
*S* = 1.07	*w* = 1/[σ^2^(*F*_o_^2^) + (0.0673*P*)^2^ + 0.272*P*] where *P* = (*F*_o_^2^ + 2*F*_c_^2^)/3
6179 reflections	(Δ/σ)_max_ < 0.001
342 parameters	Δρ_max_ = 0.34 e Å^−^^3^
0 restraints	Δρ_min_ = −0.21 e Å^−^^3^

### (Z)-2-(Ethoxymethylidene)-2,3,4,9-tetrahydro-1H-carbazol-1-one Special details

**Table:**

Geometry. All esds (except the esd in the dihedral angle between two l.s. planes) are estimated using the full covariance matrix. The cell esds are taken into account individually in the estimation of esds in distances, angles and torsion angles; correlations between esds in cell parameters are only used when they are defined by crystal symmetry. An approximate (isotropic) treatment of cell esds is used for estimating esds involving l.s. planes.

### (Z)-2-(Ethoxymethylidene)-2,3,4,9-tetrahydro-1H-carbazol-1-one Fractional atomic coordinates and isotropic or equivalent isotropic displacement parameters (Å^2^)

**Table:**

	*x*	*y*	*z*	*U*_iso_*/*U*_eq_	Occ. (<1)
C1A	0.23425 (13)	0.34202 (12)	0.25619 (10)	0.0239 (2)	
C2A	0.26411 (14)	0.24037 (13)	0.18384 (10)	0.0266 (3)	
H2A	0.354310	0.220134	0.204111	0.032*	
C3A	0.15767 (15)	0.17068 (13)	0.08202 (11)	0.0306 (3)	
H3A	0.175248	0.101102	0.031519	0.037*	
C4A	0.02347 (15)	0.20001 (14)	0.05094 (11)	0.0330 (3)	
H4AA	−0.047110	0.150424	−0.020030	0.040*	
C5A	−0.00685 (14)	0.29936 (14)	0.12181 (11)	0.0307 (3)	
H5A	−0.097523	0.318527	0.100469	0.037*	
C6A	0.09927 (13)	0.37205 (13)	0.22664 (10)	0.0255 (3)	
C7A	0.10592 (13)	0.48017 (13)	0.31754 (10)	0.0251 (3)	
C8A	−0.00457 (14)	0.55064 (15)	0.33373 (11)	0.0321 (3)	
H8A	−0.072979	0.490838	0.354650	0.039*	0.818 (5)
H8B	−0.059775	0.563188	0.264002	0.039*	0.818 (5)
H8E	−0.006593	0.621754	0.295430	0.039*	0.182 (5)
H8F	−0.100497	0.480624	0.299185	0.039*	0.182 (5)
C9A	0.06470 (18)	0.69264 (19)	0.42238 (13)	0.0296 (5)	0.818 (5)
H9A	0.101893	0.761960	0.390390	0.036*	0.818 (5)
H9B	−0.010196	0.722352	0.447143	0.036*	0.818 (5)
C9C	0.0191 (8)	0.6171 (9)	0.4461 (7)	0.034 (2)	0.182 (5)
H9E	−0.019476	0.546208	0.476290	0.041*	0.182 (5)
H9F	−0.035104	0.687433	0.449761	0.041*	0.182 (5)
C10A	0.18779 (14)	0.69280 (14)	0.52123 (11)	0.0300 (3)	
C11A	0.29143 (13)	0.61503 (12)	0.50442 (10)	0.0245 (2)	
C12A	0.24160 (13)	0.51221 (12)	0.39722 (10)	0.0242 (2)	
C13A	0.21363 (14)	0.77404 (13)	0.62250 (11)	0.0270 (3)	
H13A	0.298555	0.781728	0.681065	0.032*	
C14A	0.16297 (15)	0.93434 (15)	0.75328 (11)	0.0318 (3)	
H14A	0.254528	1.007340	0.774199	0.038*	
H14B	0.177035	0.879158	0.804658	0.038*	
C15A	0.04325 (16)	1.00019 (16)	0.75715 (13)	0.0378 (3)	
H15A	−0.045336	0.927284	0.740445	0.057*	
H15B	0.026802	1.050128	0.703074	0.057*	
H15C	0.070487	1.065960	0.830453	0.057*	
C1B	0.38735 (13)	0.64680 (12)	−0.09124 (10)	0.0247 (3)	
C2B	0.39799 (13)	0.58541 (13)	−0.19134 (11)	0.0274 (3)	
H2B	0.430439	0.640634	−0.231172	0.033*	
C3B	0.35952 (14)	0.44150 (14)	−0.23001 (11)	0.0306 (3)	
H3B	0.366065	0.397209	−0.297732	0.037*	
C4B	0.31080 (15)	0.35832 (14)	−0.17194 (12)	0.0326 (3)	
H4B	0.286954	0.259539	−0.200530	0.039*	
C5B	0.29720 (14)	0.41789 (13)	−0.07449 (11)	0.0304 (3)	
H5B	0.262638	0.361246	−0.036352	0.036*	
C6B	0.33568 (13)	0.56488 (13)	−0.03237 (10)	0.0257 (3)	
C7B	0.33468 (13)	0.65991 (13)	0.06278 (10)	0.0255 (3)	
C8B	0.29281 (15)	0.63462 (14)	0.15476 (11)	0.0306 (3)	
H8C	0.374523	0.619242	0.208837	0.037*	
H8D	0.210097	0.549638	0.126298	0.037*	
C9B	0.25147 (15)	0.75959 (14)	0.21038 (12)	0.0315 (3)	
H9C	0.152481	0.754979	0.164825	0.038*	
H9D	0.250311	0.752797	0.281954	0.038*	
C10B	0.35260 (14)	0.90015 (13)	0.22876 (11)	0.0282 (3)	
C11B	0.41058 (13)	0.92089 (13)	0.14432 (10)	0.0247 (2)	
C12B	0.38651 (13)	0.79301 (13)	0.05942 (10)	0.0242 (2)	
C13B	0.38397 (15)	1.01030 (14)	0.31712 (11)	0.0318 (3)	
H13B	0.446278	1.097120	0.325510	0.038*	
C14B	0.3672 (2)	1.12403 (16)	0.48516 (14)	0.0499 (4)	
H14C	0.346185	1.202473	0.458539	0.060*	
H14D	0.471470	1.150291	0.529861	0.060*	
C15B	0.2787 (3)	1.09123 (18)	0.55176 (16)	0.0614 (6)	
H15D	0.301796	1.173573	0.614879	0.092*	
H15E	0.175890	1.065326	0.506436	0.092*	
H15F	0.300560	1.013401	0.577408	0.092*	
N1A	0.31972 (11)	0.42848 (11)	0.36028 (9)	0.0249 (2)	
H1A	0.4135 (18)	0.4321 (15)	0.3973 (13)	0.030*	
N1B	0.41918 (11)	0.78529 (11)	−0.03362 (9)	0.0252 (2)	
H1B	0.4562 (16)	0.8590 (17)	−0.0527 (12)	0.030*	
O1A	0.41162 (9)	0.63464 (9)	0.57565 (7)	0.0289 (2)	
O2A	0.12090 (10)	0.84483 (10)	0.64230 (8)	0.0328 (2)	
O1B	0.47513 (10)	1.03698 (9)	0.14503 (8)	0.0290 (2)	
O2B	0.32788 (12)	0.99881 (10)	0.39402 (8)	0.0380 (2)	

### (Z)-2-(Ethoxymethylidene)-2,3,4,9-tetrahydro-1H-carbazol-1-one Atomic displacement parameters (Å^2^)

**Table:**

	*U*^11^	*U*^22^	*U*^33^	*U*^12^	*U*^13^	*U*^23^
C1A	0.0238 (6)	0.0217 (5)	0.0243 (6)	0.0029 (4)	0.0075 (5)	0.0086 (5)
C2A	0.0276 (6)	0.0249 (6)	0.0275 (6)	0.0058 (5)	0.0108 (5)	0.0088 (5)
C3A	0.0355 (7)	0.0250 (6)	0.0277 (6)	0.0045 (5)	0.0114 (5)	0.0057 (5)
C4A	0.0322 (7)	0.0312 (7)	0.0260 (6)	0.0025 (5)	0.0039 (5)	0.0062 (5)
C5A	0.0259 (6)	0.0306 (6)	0.0307 (7)	0.0051 (5)	0.0051 (5)	0.0102 (5)
C6A	0.0246 (6)	0.0247 (6)	0.0271 (6)	0.0048 (5)	0.0087 (5)	0.0107 (5)
C7A	0.0227 (6)	0.0250 (6)	0.0271 (6)	0.0054 (5)	0.0078 (5)	0.0102 (5)
C8A	0.0242 (6)	0.0362 (7)	0.0333 (7)	0.0117 (5)	0.0073 (5)	0.0080 (6)
C9A	0.0289 (8)	0.0341 (10)	0.0294 (8)	0.0154 (7)	0.0109 (6)	0.0108 (7)
C9C	0.022 (3)	0.031 (4)	0.043 (5)	0.007 (3)	0.009 (3)	0.007 (3)
C10A	0.0273 (6)	0.0328 (7)	0.0301 (7)	0.0121 (5)	0.0098 (5)	0.0086 (5)
C11A	0.0246 (6)	0.0242 (6)	0.0273 (6)	0.0075 (5)	0.0108 (5)	0.0108 (5)
C12A	0.0239 (6)	0.0247 (6)	0.0256 (6)	0.0078 (5)	0.0095 (5)	0.0095 (5)
C13A	0.0262 (6)	0.0262 (6)	0.0318 (6)	0.0097 (5)	0.0118 (5)	0.0114 (5)
C14A	0.0361 (7)	0.0335 (7)	0.0281 (7)	0.0131 (6)	0.0142 (6)	0.0082 (5)
C15A	0.0407 (8)	0.0395 (8)	0.0386 (8)	0.0162 (6)	0.0215 (6)	0.0093 (6)
C1B	0.0213 (5)	0.0229 (6)	0.0287 (6)	0.0059 (4)	0.0083 (5)	0.0076 (5)
C2B	0.0242 (6)	0.0278 (6)	0.0307 (6)	0.0069 (5)	0.0116 (5)	0.0085 (5)
C3B	0.0278 (6)	0.0300 (6)	0.0327 (7)	0.0099 (5)	0.0117 (5)	0.0052 (5)
C4B	0.0329 (7)	0.0228 (6)	0.0401 (7)	0.0082 (5)	0.0126 (6)	0.0069 (5)
C5B	0.0306 (7)	0.0245 (6)	0.0372 (7)	0.0077 (5)	0.0128 (5)	0.0116 (5)
C6B	0.0231 (6)	0.0247 (6)	0.0300 (6)	0.0071 (5)	0.0095 (5)	0.0104 (5)
C7B	0.0237 (6)	0.0254 (6)	0.0289 (6)	0.0080 (5)	0.0099 (5)	0.0103 (5)
C8B	0.0362 (7)	0.0273 (6)	0.0330 (7)	0.0087 (5)	0.0165 (6)	0.0134 (5)
C9B	0.0355 (7)	0.0313 (7)	0.0337 (7)	0.0090 (5)	0.0186 (6)	0.0132 (6)
C10B	0.0313 (6)	0.0288 (6)	0.0295 (6)	0.0100 (5)	0.0141 (5)	0.0132 (5)
C11B	0.0239 (6)	0.0255 (6)	0.0267 (6)	0.0087 (5)	0.0099 (5)	0.0099 (5)
C12B	0.0227 (6)	0.0263 (6)	0.0258 (6)	0.0080 (5)	0.0100 (5)	0.0096 (5)
C13B	0.0390 (7)	0.0319 (7)	0.0300 (7)	0.0112 (6)	0.0162 (6)	0.0141 (5)
C14B	0.0864 (13)	0.0271 (7)	0.0395 (9)	0.0084 (8)	0.0346 (9)	0.0074 (6)
C15B	0.1133 (17)	0.0330 (8)	0.0491 (10)	0.0139 (9)	0.0531 (11)	0.0072 (7)
N1A	0.0227 (5)	0.0255 (5)	0.0245 (5)	0.0074 (4)	0.0066 (4)	0.0064 (4)
N1B	0.0269 (5)	0.0228 (5)	0.0272 (5)	0.0055 (4)	0.0124 (4)	0.0082 (4)
O1A	0.0246 (4)	0.0310 (5)	0.0272 (5)	0.0094 (4)	0.0063 (4)	0.0055 (4)
O2A	0.0329 (5)	0.0359 (5)	0.0311 (5)	0.0155 (4)	0.0136 (4)	0.0065 (4)
O1B	0.0328 (5)	0.0243 (4)	0.0313 (5)	0.0056 (4)	0.0150 (4)	0.0090 (4)
O2B	0.0569 (6)	0.0296 (5)	0.0326 (5)	0.0099 (4)	0.0253 (5)	0.0091 (4)

### (Z)-2-(Ethoxymethylidene)-2,3,4,9-tetrahydro-1H-carbazol-1-one Geometric parameters (Å, º)

**Table:**

C1A—N1A	1.3751 (16)	C15A—H15B	0.9800
C1A—C2A	1.4003 (17)	C15A—H15C	0.9800
C1A—C6A	1.4200 (17)	C1B—N1B	1.3727 (16)
C2A—C3A	1.3798 (18)	C1B—C2B	1.3981 (18)
C2A—H2A	0.9500	C1B—C6B	1.4218 (17)
C3A—C4A	1.410 (2)	C2B—C3B	1.3795 (18)
C3A—H3A	0.9500	C2B—H2B	0.9500
C4A—C5A	1.377 (2)	C3B—C4B	1.409 (2)
C4A—H4AA	0.9500	C3B—H3B	0.9500
C5A—C6A	1.4090 (18)	C4B—C5B	1.376 (2)
C5A—H5A	0.9500	C4B—H4B	0.9500
C6A—C7A	1.4235 (17)	C5B—C6B	1.4114 (17)
C7A—C12A	1.3843 (17)	C5B—H5B	0.9500
C7A—C8A	1.4887 (17)	C6B—C7B	1.4241 (17)
C8A—C9C	1.434 (8)	C7B—C12B	1.3855 (17)
C8A—C9A	1.526 (2)	C7B—C8B	1.4932 (18)
C8A—H8A	0.9900	C8B—C9B	1.5279 (19)
C8A—H8B	0.9900	C8B—H8C	0.9900
C8A—H8E	0.9900	C8B—H8D	0.9900
C8A—H8F	0.9900	C9B—C10B	1.5205 (18)
C9A—C10A	1.524 (2)	C9B—H9C	0.9900
C9A—H9A	0.9900	C9B—H9D	0.9900
C9A—H9B	0.9900	C10B—C13B	1.3423 (19)
C9C—C10A	1.620 (7)	C10B—C11B	1.4748 (17)
C9C—H9E	0.9900	C11B—O1B	1.2432 (15)
C9C—H9F	0.9900	C11B—C12B	1.4487 (17)
C10A—C13A	1.3409 (18)	C12B—N1B	1.3849 (16)
C10A—C11A	1.4761 (17)	C13B—O2B	1.3471 (16)
C11A—O1A	1.2455 (15)	C13B—H13B	0.9500
C11A—C12A	1.4483 (17)	C14B—O2B	1.4452 (17)
C12A—N1A	1.3853 (16)	C14B—C15B	1.503 (2)
C13A—O2A	1.3457 (15)	C14B—H14C	0.9900
C13A—H13A	0.9500	C14B—H14D	0.9900
C14A—O2A	1.4446 (16)	C15B—H15D	0.9800
C14A—C15A	1.5078 (19)	C15B—H15E	0.9800
C14A—H14A	0.9900	C15B—H15F	0.9800
C14A—H14B	0.9900	N1A—H1A	0.919 (16)
C15A—H15A	0.9800	N1B—H1B	0.908 (17)
			
N1A—C1A—C2A	130.08 (12)	C14A—C15A—H15C	109.5
N1A—C1A—C6A	108.42 (11)	H15A—C15A—H15C	109.5
C2A—C1A—C6A	121.49 (11)	H15B—C15A—H15C	109.5
C3A—C2A—C1A	117.54 (12)	N1B—C1B—C2B	129.84 (12)
C3A—C2A—H2A	121.2	N1B—C1B—C6B	108.51 (11)
C1A—C2A—H2A	121.2	C2B—C1B—C6B	121.65 (11)
C2A—C3A—C4A	121.74 (12)	C3B—C2B—C1B	117.40 (12)
C2A—C3A—H3A	119.1	C3B—C2B—H2B	121.3
C4A—C3A—H3A	119.1	C1B—C2B—H2B	121.3
C5A—C4A—C3A	121.04 (12)	C2B—C3B—C4B	121.89 (12)
C5A—C4A—H4AA	119.5	C2B—C3B—H3B	119.1
C3A—C4A—H4AA	119.5	C4B—C3B—H3B	119.1
C4A—C5A—C6A	118.68 (12)	C5B—C4B—C3B	121.05 (12)
C4A—C5A—H5A	120.7	C5B—C4B—H4B	119.5
C6A—C5A—H5A	120.7	C3B—C4B—H4B	119.5
C5A—C6A—C1A	119.51 (12)	C4B—C5B—C6B	118.62 (12)
C5A—C6A—C7A	133.63 (12)	C4B—C5B—H5B	120.7
C1A—C6A—C7A	106.84 (11)	C6B—C5B—H5B	120.7
C12A—C7A—C6A	106.75 (11)	C5B—C6B—C1B	119.36 (12)
C12A—C7A—C8A	122.71 (11)	C5B—C6B—C7B	133.85 (12)
C6A—C7A—C8A	130.53 (11)	C1B—C6B—C7B	106.79 (11)
C9C—C8A—C7A	114.4 (3)	C12B—C7B—C6B	106.63 (11)
C7A—C8A—C9A	111.43 (11)	C12B—C7B—C8B	122.42 (11)
C7A—C8A—H8A	109.3	C6B—C7B—C8B	130.93 (11)
C9A—C8A—H8A	109.3	C7B—C8B—C9B	110.29 (10)
C7A—C8A—H8B	109.3	C7B—C8B—H8C	109.6
C9A—C8A—H8B	109.3	C9B—C8B—H8C	109.6
H8A—C8A—H8B	108.0	C7B—C8B—H8D	109.6
C9C—C8A—H8E	108.7	C9B—C8B—H8D	109.6
C7A—C8A—H8E	108.7	H8C—C8B—H8D	108.1
C9C—C8A—H8F	108.7	C10B—C9B—C8B	113.84 (11)
C7A—C8A—H8F	108.7	C10B—C9B—H9C	108.8
H8E—C8A—H8F	107.6	C8B—C9B—H9C	108.8
C10A—C9A—C8A	113.52 (13)	C10B—C9B—H9D	108.8
C10A—C9A—H9A	108.9	C8B—C9B—H9D	108.8
C8A—C9A—H9A	108.9	H9C—C9B—H9D	107.7
C10A—C9A—H9B	108.9	C13B—C10B—C11B	118.17 (12)
C8A—C9A—H9B	108.9	C13B—C10B—C9B	121.83 (12)
H9A—C9A—H9B	107.7	C11B—C10B—C9B	119.85 (11)
C8A—C9C—C10A	113.2 (5)	O1B—C11B—C12B	122.29 (11)
C8A—C9C—H9E	108.9	O1B—C11B—C10B	123.70 (11)
C10A—C9C—H9E	108.9	C12B—C11B—C10B	114.01 (11)
C8A—C9C—H9F	108.9	N1B—C12B—C7B	110.03 (11)
C10A—C9C—H9F	108.9	N1B—C12B—C11B	125.25 (11)
H9E—C9C—H9F	107.8	C7B—C12B—C11B	124.63 (11)
C13A—C10A—C11A	118.71 (12)	C10B—C13B—O2B	120.58 (12)
C13A—C10A—C9A	121.40 (12)	C10B—C13B—H13B	119.7
C11A—C10A—C9A	119.50 (12)	O2B—C13B—H13B	119.7
C13A—C10A—C9C	115.3 (3)	O2B—C14B—C15B	106.50 (13)
C11A—C10A—C9C	116.8 (3)	O2B—C14B—H14C	110.4
O1A—C11A—C12A	122.17 (11)	C15B—C14B—H14C	110.4
O1A—C11A—C10A	123.55 (11)	O2B—C14B—H14D	110.4
C12A—C11A—C10A	114.28 (11)	C15B—C14B—H14D	110.4
C7A—C12A—N1A	109.92 (11)	H14C—C14B—H14D	108.6
C7A—C12A—C11A	124.54 (11)	C14B—C15B—H15D	109.5
N1A—C12A—C11A	125.50 (11)	C14B—C15B—H15E	109.5
C10A—C13A—O2A	120.91 (12)	H15D—C15B—H15E	109.5
C10A—C13A—H13A	119.5	C14B—C15B—H15F	109.5
O2A—C13A—H13A	119.5	H15D—C15B—H15F	109.5
O2A—C14A—C15A	107.13 (11)	H15E—C15B—H15F	109.5
O2A—C14A—H14A	110.3	C1A—N1A—C12A	108.06 (10)
C15A—C14A—H14A	110.3	C1A—N1A—H1A	125.6 (9)
O2A—C14A—H14B	110.3	C12A—N1A—H1A	126.3 (9)
C15A—C14A—H14B	110.3	C1B—N1B—C12B	108.04 (10)
H14A—C14A—H14B	108.5	C1B—N1B—H1B	126.0 (10)
C14A—C15A—H15A	109.5	C12B—N1B—H1B	125.9 (10)
C14A—C15A—H15B	109.5	C13A—O2A—C14A	116.18 (10)
H15A—C15A—H15B	109.5	C13B—O2B—C14B	116.12 (11)
			
N1A—C1A—C2A—C3A	−178.43 (12)	C1B—C2B—C3B—C4B	0.20 (19)
C6A—C1A—C2A—C3A	0.44 (18)	C2B—C3B—C4B—C5B	1.1 (2)
C1A—C2A—C3A—C4A	0.13 (19)	C3B—C4B—C5B—C6B	−1.1 (2)
C2A—C3A—C4A—C5A	−0.4 (2)	C4B—C5B—C6B—C1B	−0.13 (19)
C3A—C4A—C5A—C6A	0.1 (2)	C4B—C5B—C6B—C7B	179.41 (13)
C4A—C5A—C6A—C1A	0.42 (19)	N1B—C1B—C6B—C5B	−179.17 (11)
C4A—C5A—C6A—C7A	178.64 (13)	C2B—C1B—C6B—C5B	1.50 (18)
N1A—C1A—C6A—C5A	178.36 (11)	N1B—C1B—C6B—C7B	1.17 (13)
C2A—C1A—C6A—C5A	−0.73 (18)	C2B—C1B—C6B—C7B	−178.17 (11)
N1A—C1A—C6A—C7A	−0.29 (13)	C5B—C6B—C7B—C12B	179.56 (14)
C2A—C1A—C6A—C7A	−179.38 (11)	C1B—C6B—C7B—C12B	−0.85 (13)
C5A—C6A—C7A—C12A	−178.05 (14)	C5B—C6B—C7B—C8B	1.1 (2)
C1A—C6A—C7A—C12A	0.33 (13)	C1B—C6B—C7B—C8B	−179.30 (13)
C5A—C6A—C7A—C8A	3.0 (2)	C12B—C7B—C8B—C9B	24.95 (17)
C1A—C6A—C7A—C8A	−178.58 (13)	C6B—C7B—C8B—C9B	−156.82 (13)
C12A—C7A—C8A—C9C	−20.1 (4)	C7B—C8B—C9B—C10B	−43.16 (16)
C6A—C7A—C8A—C9C	158.7 (4)	C8B—C9B—C10B—C13B	−145.39 (13)
C12A—C7A—C8A—C9A	22.51 (18)	C8B—C9B—C10B—C11B	39.10 (17)
C6A—C7A—C8A—C9A	−158.73 (14)	C13B—C10B—C11B—O1B	−7.15 (19)
C7A—C8A—C9A—C10A	−40.90 (18)	C9B—C10B—C11B—O1B	168.52 (12)
C7A—C8A—C9C—C10A	38.7 (6)	C13B—C10B—C11B—C12B	172.56 (12)
C8A—C9A—C10A—C13A	−146.49 (14)	C9B—C10B—C11B—C12B	−11.77 (17)
C8A—C9A—C10A—C11A	40.82 (19)	C6B—C7B—C12B—N1B	0.24 (14)
C8A—C9C—C10A—C13A	170.2 (4)	C8B—C7B—C12B—N1B	178.85 (11)
C8A—C9C—C10A—C11A	−43.3 (7)	C6B—C7B—C12B—C11B	−176.44 (11)
C13A—C10A—C11A—O1A	−10.2 (2)	C8B—C7B—C12B—C11B	2.17 (19)
C9A—C10A—C11A—O1A	162.73 (14)	O1B—C11B—C12B—N1B	−6.12 (19)
C9C—C10A—C11A—O1A	−155.4 (4)	C10B—C11B—C12B—N1B	174.16 (11)
C13A—C10A—C11A—C12A	169.61 (12)	O1B—C11B—C12B—C7B	170.06 (12)
C9A—C10A—C11A—C12A	−17.50 (18)	C10B—C11B—C12B—C7B	−9.65 (18)
C9C—C10A—C11A—C12A	24.3 (4)	C11B—C10B—C13B—O2B	176.66 (12)
C6A—C7A—C12A—N1A	−0.25 (14)	C9B—C10B—C13B—O2B	1.1 (2)
C8A—C7A—C12A—N1A	178.76 (11)	C2A—C1A—N1A—C12A	179.12 (12)
C6A—C7A—C12A—C11A	−178.34 (11)	C6A—C1A—N1A—C12A	0.14 (13)
C8A—C7A—C12A—C11A	0.67 (19)	C7A—C12A—N1A—C1A	0.07 (14)
O1A—C11A—C12A—C7A	175.85 (12)	C11A—C12A—N1A—C1A	178.14 (11)
C10A—C11A—C12A—C7A	−3.92 (18)	C2B—C1B—N1B—C12B	178.23 (12)
O1A—C11A—C12A—N1A	−1.9 (2)	C6B—C1B—N1B—C12B	−1.03 (13)
C10A—C11A—C12A—N1A	178.28 (11)	C7B—C12B—N1B—C1B	0.49 (14)
C11A—C10A—C13A—O2A	−178.70 (11)	C11B—C12B—N1B—C1B	177.15 (11)
C9A—C10A—C13A—O2A	8.6 (2)	C10A—C13A—O2A—C14A	−177.10 (12)
C9C—C10A—C13A—O2A	−32.9 (4)	C15A—C14A—O2A—C13A	−178.88 (11)
N1B—C1B—C2B—C3B	179.32 (12)	C10B—C13B—O2B—C14B	−179.32 (14)
C6B—C1B—C2B—C3B	−1.50 (18)	C15B—C14B—O2B—C13B	173.13 (15)

### (Z)-2-(Ethoxymethylidene)-2,3,4,9-tetrahydro-1H-carbazol-1-one Hydrogen-bond geometry (Å, º)

*Cg*5 and *Cg*2 are the centroids of the (N1*B*/C1*B*/C6*B*/C7*B*/C12*B*) pyrrole ring and (C1*A*–C6*A*) benzene rings respectively.

**Table:**

*D*—H···*A*	*D*—H	H···*A*	*D*···*A*	*D*—H···*A*
C2*A*—H2*A*···O1*B*^i^	0.95	2.54	3.3335 (16)	141
C2*B*—H2*B*···O1*A*^ii^	0.95	2.54	3.3457 (16)	142
N1*A*—H1*A*···O1*A*^iii^	0.919 (16)	1.985 (16)	2.8462 (14)	155.4 (13)
N1*B*—H1*B*···O1*B*^iv^	0.908 (17)	2.017 (17)	2.8807 (14)	158.5 (14)
C5*A*—H5*A*···*Cg*5^v^	0.95	2.66	3.5939 (16)	166
C8*B*—H8*D*···*Cg*2	0.99	2.97	3.7871 (16)	141
C15*A*—H15*A*···*Cg*2^vi^	0.98	2.89	3.6449 (18)	134

Symmetry codes: (i) *x*, *y*−1, *z*; (ii) *x*, *y*, *z*−1; (iii) −*x*+1, −*y*+1, −*z*+1; (iv) −*x*+1, −*y*+2, −*z*; (v) −*x*, −*y*+1, −*z*; (vi) −*x*, −*y*+1, −*z*+1.
